# Real-World Outcomes of a Rhythm Control Strategy for Atrial Fibrillation Patients with Reduced Left Ventricular Ejection Fraction (<50%)

**DOI:** 10.3390/jcm13113285

**Published:** 2024-06-02

**Authors:** Ji-Hoon Choi, Chang Hee Kwon

**Affiliations:** Department of Internal Medicine, Division of Cardiology, Konkuk University Medical Center, Konkuk University School of Medicine, Seoul 05030, Republic of Korea; 20220201@kuh.ac.kr

**Keywords:** atrial fibrillation, brain natriuretic peptide, left ventricular ejection fraction, rhythm control

## Abstract

**Background/Objectives**: The effectiveness of a rhythm control strategy in patients with atrial fibrillation (AF) and reduced left ventricular ejection fraction (LVEF < 50%) in real-world practice remains uncertain. Our objective was to evaluate the real-world outcomes of a rhythm control strategy in patients with AF and reduced LVEF, focusing on changes in LV systolic function and brain natriuretic peptide (BNP) levels. **Methods**: The study retrospectively reviewed the medical records of 80 patients with concurrent AF and reduced LVEF who underwent rhythm control therapy between March 2015 and December 2021. **Results**: The patients had an average age of 63.6 years and an initial LVEF of 34.3%. Sinus rhythm was restored using anti-arrhythmic drugs (38, 47.5%) or electrical cardioversion (42, 52.5%). Over a follow-up period of 53.0 months, AF recurred in 65% of patients, with 57.7% undergoing catheter ablation. Significant improvements were noted in LVEF (from 34.3% to 55.1%, *p* < 0.001) and BNP levels (from 752 pg/mL to 72 pg/mL, *p* < 0.001) at the last follow-up. Nearly all patients (97.5%) continued with the rhythm control strategy during the follow-up period. **Conclusions**: In real-world settings, a rhythm control strategy proves to be feasible and effective for improving LVEF and reducing BNP levels in AF patients with reduced LVEF.

## 1. Introduction

Atrial fibrillation (AF) is the most common cardiac arrhythmia and is associated with significant comorbidities [1,2]. Notably, heart failure (HF), a severe outcome related to AF, represents a major cause of mortality and morbidity [3]. Given that AF can be both a cause and consequence of HF, the co-existence of these two conditions is frequently encountered in clinical practice [4,5]. The prevalence of HF among patients with AF ranges from 20 to 30%, while AF is reported in 30–50% of patients with HF [4,6,7]. Consequently, patients with both AF and HF are at a higher risk of cardiovascular complications compared to those with only one of the conditions [8,9].

Managing AF in patients with reduced left ventricular ejection fraction (LVEF) poses unique challenges. The selection of anti-arrhythmic drugs (AADs) is constrained by their potential proarrhythmic risk and poor tolerance among these patients. Furthermore, there are no clinical trials that have definitively shown the superiority of an AADs-based rhythm control strategy over rate control [10,11,12,13]. However, the EAST-AFNET4 and CASTLE-AF trials have demonstrated the clinical benefit of an ablation-based rhythm control strategy compared to rate control in patients with AF and reduced LVEF [14,15]. Moreover, in patients with symptomatic AF and end-stage HF, combining catheter ablation with guideline-directed medical therapy (GDMT) was linked to a reduced risk of a composite outcome, including death from any cause, implantation of a left ventricular assist device, or urgent heart transplantation, compared to medical therapy alone [16]. Despite this, many patients are not considered candidates for catheter ablation at their initial presentation and typically begin their treatment with an AADs-based rhythm control strategy, including early attempts at electrical cardioversion, and may consider catheter ablation if AF recurs. A recent study has shown that a pharmacological rhythm control strategy is not superior to a rate control strategy in providing a survival advantage for recently hospitalized patients with AF and comorbid HF [17]. The cardiovascular benefits of a rhythm control strategy in real-world setting remain underexplored. Thus, our study aims to examine the real-world outcomes of an early rhythm control strategy in patients with AF and reduced LVEF, with a particular focus on the recovery of LV systolic function. 

## 2. Materials and Methods

### 2.1. Study Patients

A total of 80 patients, aged 18 years and older, with concurrent AF and reduced LVEF (<50%), who received rhythm control therapy at our institution (Konkuk University Medical Center, Seoul, Republic of Korea) between March 2015 and December 2021, were consecutively included in this retrospective study. These patients did not present with secondary causes of AF, such as surgery, metabolic disorders (primarily hyperthyroidism), infection, alcohol, and substance use, at the time of diagnosis, and there were no indications of myocardial infarction or specific cardiomyopathy as a cause of the reduced LVEF. Fifty patients (62.5%) were diagnosed during hospital admission, while thirty patients (37.5%) were diagnosed at outpatient clinics. Clinical and echocardiographic variables, along with serum brain natriuretic peptide (BNP) levels, were collected. Data collection adhered to the principles of the Declaration of Helsinki (2013) and Good Clinical Practices. The research protocol was reviewed and approved by the Institutional Review Board of Konkuk University Medical Center (protocol no. KUMC 2023-02-021) on 13 February 2023. Information was retrieved retrospectively in a de-identified manner, thereby waiving the requirement for informed consent.

### 2.2. Patient Follow-Up

All patients received GDMT for HF, which included a beta-blocker, an angiotensin-converting enzyme inhibitor or angiotensin receptor blocker, and a mineralocorticoid receptor antagonist unless contraindications to these drugs existed. HF symptoms, such as dyspnea, dyspnea on exertion, or pitting edema, were managed with diuretics. Non-vitamin K antagonist oral anticoagulants were administered to all patients for at least three weeks prior to undergoing chemical or electrical cardioversion. 

Initially, amiodarone was used as the first-line AADs for most patients presenting with HF. It was then replaced with other AADs (propafenone, flecainide, dronedarone, or sotalol) based on the improvement of LVEF and/or the presence of structural heart disease. Patients were followed up with after 2 weeks or 1 month until the stabilization of HF symptoms was achieved. After stabilization, follow-ups were conducted every 3 months, or patients visited the clinic if they experienced any symptoms related to AF or HF. 

The recurrence of AF was assessed through 12-lead electrocardiography at each clinic visit, or through Holter monitoring and/or a wearable patch if patients reported symptoms indicative of AF. The clinical recurrence of AF was identified as any electrocardiographically documented atrial tachyarrhythmias, including AF, atrial flutter, or atypical atrial flutter, lasting more than 30 s. Catheter ablation was recommended for patients experiencing any recurrence of atrial tachyarrhythmias after taking AADs for at least 6 weeks, following the guideline of Korean health insurance. Additionally, when patients developed sick sinus syndrome after the restoration of sinus rhythm or exhibited symptomatic adverse effects from AADs, catheter ablation was performed for the rhythm control of AF. If patients and/or their guardians did consent to catheter ablation, we recommended electrical cardioversion. When they chose not to continue with the rhythm control strategy for AF, we abandoned rhythm control in favor of managing HF symptoms, including rate control.

### 2.3. Echocardiographic Examination

Echocardiographic profiles were measured at the echocardiographic laboratory of our institution following a protocol established by the American Society of Echocardiography. All echocardiographic examinations were evaluated and interpreted by a cardiologist specializing in echocardiography.

The M-mode parasternal long-axis view at the end of the LV systole was utilized to measure the left atrial (LA) diameter. The LA volume index was measured using the biplane area–length method through the apical four-chamber and apical two-chamber view at the ventricular end-systole, and was indexed to the calculated body surface area using the Du Bois formula. The LVEF was assessed using the biplane Simpson method, employing the end-diastolic and end-systolic apical 4- and 2-chamber views to estimate LV volume and calculate the EF.

### 2.4. Study Outcomes

The primary outcomes of interest in this study were the changes in LVEF and BNP levels following rhythm control therapy. Baseline echocardiography was performed at the time of AF diagnosis. Follow-up echocardiography was conducted at least three months after starting rhythm control therapy and continued until complete recovery of LV systolic function was observed (LVEF ≥ 50%). After recovery of LVEF to 50% or above, annual examinations were performed. Serum BNP levels were checked at baseline and at every clinic visit until they reached the normal range. Thereafter, BNP tests were conducted every 6 months, at the annual follow-up, or when the patient’s condition changed. Secondary outcomes of interest included the number of patients requiring catheter ablation as part of the rhythm control strategy and the number of patients continuing with the rhythm control strategy. 

### 2.5. Statistical Analysis

Data analysis was performed using SPSS statistical software (version 25; SPSS Inc., Chicago, IL, USA). Continuous variables are reported as mean values ± standard deviation, while categorical variables are reported as the number of patients (percentage). Comparisons of serial changes in LVEF and BNP levels were conducted using the paired *t*-test. This statistical approach allowed for the assessment of significant differences in LVEF and BNP values measured at different time points during the study, thereby evaluating the impact of rhythm control therapy over time. The independent sample *t*-test and paired *t*-test were performed for comparison of the two groups. All statistical tests were two-tailed, and a *p*-value of <0.05 was considered statistically significant.

## 3. Results

### 3.1. Baseline Characteristics and Outcome of Rhythm Control

**Figure 1:**
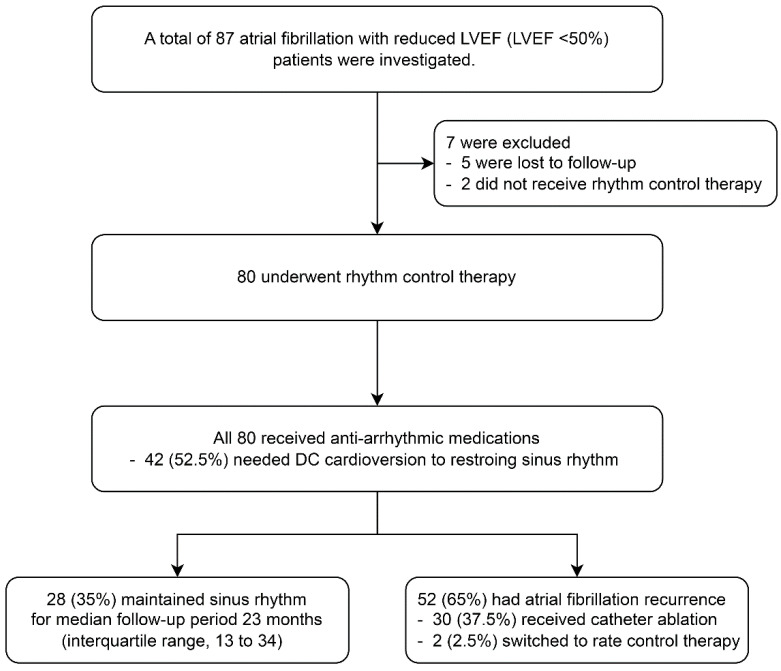
Patient flow diagram according to clinical course.

Between March 2015 and December 2021, a total of 87 patients with AF and reduced LVEF were identified. Seven patients were excluded from this study due to loss of follow-up (five patients) and not receiving rhythm control therapy (two patients), resulting in 80 patients being included in the analysis (Figure 1).

Table 1 presents the baseline characteristics of study patients. The average age was 64 years, with a predominance of male patients (65/80, 81.3%). At the initial evaluation, the majority (73/80, 91.3%) had persistent AF, and 15 (18.8%) were categorized as having longstanding persistent AF (defined as persistent AF lasting longer than one year). A minority had a history of myocardial infarction (1, 1.3%) or had undergone percutaneous coronary intervention (4, 5.0%). The mean CHA_2_DS_2_VASc score was 2.4, with 32 (40.0%) patients scoring 3 or higher. The mean LA volume index was 41.8 ± 14.7 mL/m^2^ with 67 (83.8%) patients meeting the criteria for LA enlargement (LA volume index ≥ 34 mL/m^2^).

The patient flow diagram, depicted in Figure 1, outlines the clinical course of participants. All 80 patients were treated with AADs, and 42 (52.5%) required electrical cardioversion to restore sinus rhythm. Over a follow-up period of 53.0 ± 25.5 months, AF recurred in 52 patients (65.0%), with a median recurrence time of 7.7 months (interquartile range 2.4 to 17.0 months). Among those experiencing AF recurrence, 30 patients (57.7%) underwent catheter ablation as part of the rhythm control strategy. Only two patients (2.5%) failed to maintain sinus rhythm and were transitioned to rate control therapy. Seventy (87.5%) patients continued their AADs medication until their last follow-up time. Notably, during the follow-up period, there were two cases of death (one from unknown cause and the other from pneumonia), one case of hospitalization for HF, and two cases of stroke. Additionally, there was no need for implantable cardioverter–defibrillator placement or cardiac resynchronization therapy.

### 3.2. Improvement in Echocardiographic Parameters and Brain Natriuretic Peptide

**Figure 2:**
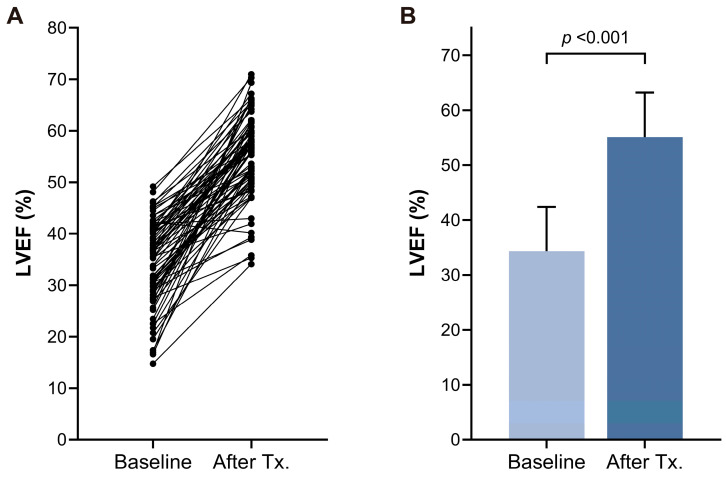
Change of left ventricular ejection fraction (LVEF) represented as an individual plot (**A**) and bar graph (**B**) at baseline and after rhythm control therapy. Tx = therapy.

At initial presentation, the mean LVEF was 34.3 ± 8.1%, with severe LV systolic dysfunction (LVEF < 40%) detected in approximately two-thirds of patients (55/80, 68.8%). As depicted in Figure 2A, except for one patient (1/80, 1.3%), all participants showed an improvement in LVEF within one year following rhythm control therapy (Figure 2B). The mean improvement in LVEF was 20.8 ± 9.9% (baseline vs. last follow-up; 34.3 ± 8.1% vs. 55.1 ± 8.1%; *p* < 0.001). Complete recovery of LV systolic function (LVEF ≥ 50%) was achieved in 61 patients (76.3%) within one year, and in 65 patients (81.3%) by the last follow-up after undergoing rhythm control therapy.

**Figure 3:**
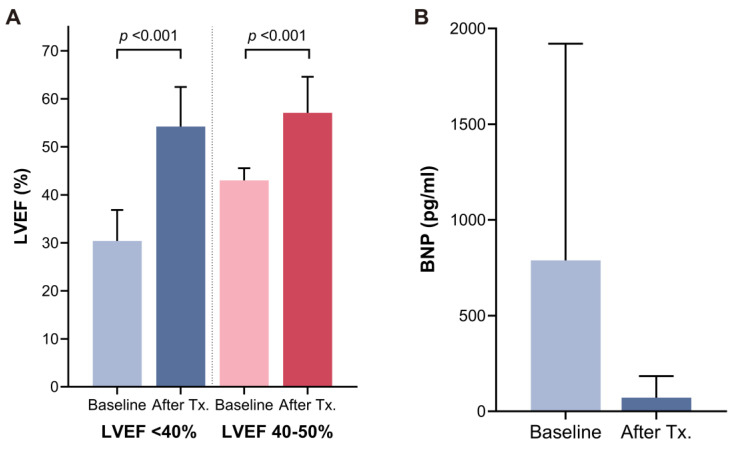
(**A**). Subgroup analysis of change of left ventricular ejection fraction (LVEF) according to baseline left ventricular systolic function. (**B**). Change of brain natriuretic peptide (BNP) at baseline and after rhythm control therapy. Tx = therapy.

**Figure 4:**
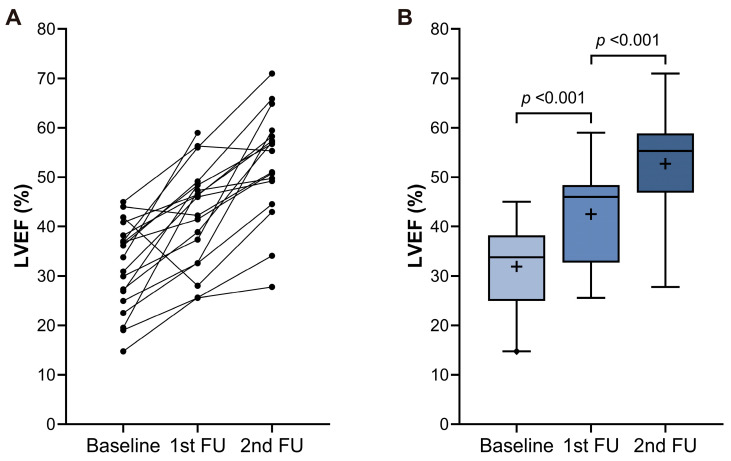
Change of left ventricular ejection fraction (LVEF) in patients who underwent serial echocardiography before (only guideline-directed medical therapy for heart failure) and after rhythm control therapy represented as an individual plot (**A**) and bar graph (**B**). LVEF showed sequential improvement after GDMT for HF (1st follow-up) and rhythm control therapy (2nd follow-up). FU = follow-up. In Figure B, the height of the box indicates the interquartile range (IQR), the horizontal bar within the box indicates the median, the cross within the box indicates the mean, and the whiskers indicate 1.5 times the IQR.

**Figure 5:**
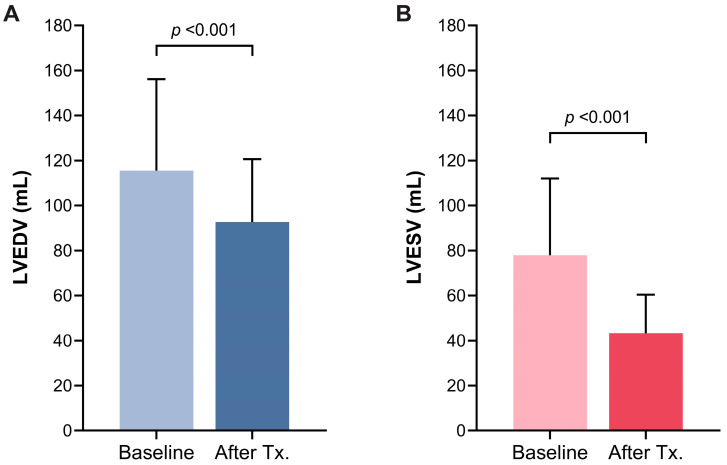
Change of left ventricular end-diastolic volume (**A**) and end-systolic volume (**B**) at baseline and after rhythm control therapy.

**Figure 6:**
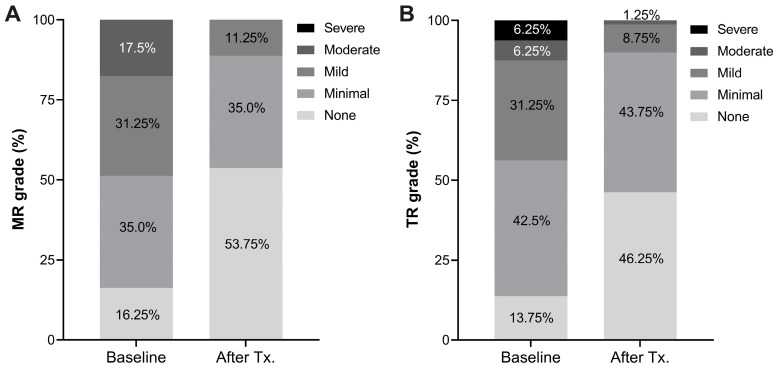
Change of mitral (**A**) and tricuspid (**B**) regurgitation grade at baseline and after rhythm control therapy.

Subgroup analysis demonstrated consistent improvement of LVEF across different baseline levels (from 30.4 ± 6.5% to 54.2 ± 8.3% for those with LVEF < 40%, and form 43.0 ± 2.6% to 57.1 ± 7.5% for those with LVEF 40–50%) (Figure 3A). A significant decrease in the level of BNP was observed after rhythm control therapy compared with baseline (baseline vs. last follow-up; 752 ± 1039 pg/mL vs. 72 ± 114 pg/mL; *p* < 0.001) (Figure 3B). Seventeen patients (21.3%) who initiated GDMT for HF underwent serial echocardiography before and after rhythm control therapy. In a subgroup analysis of these patients, LVEF showed sequential improvement after GDMT for HF (1st follow-up) and rhythm control therapy (2nd follow-up) (baseline vs. after GDMT for HF, vs. after rhythm control; 32.7 ± 8.5% vs. 45.2 ± 9.7% vs. 56.3 ± 10.0%) (Figure 4). Moreover, left ventricular end-diastolic and end-systolic volumes exhibited significant decreases before and after rhythm control treatment (Figure 5). Fourteen patients (17.5%) presented with moderate mitral regurgitation initially, yet all demonstrated mild or less regurgitation upon the last follow-up echocardiography (Figure 6A). Additionally, moderate and severe tricuspid regurgitation were identified in 10 patients (12.5%). Following rhythm control treatment, only one patient (1.3%) exhibited moderate tricuspid regurgitation (Figure 6B).

### 3.3. Comparison of Baseline Characteristics and Outcome of Rhythm Control between Patients with or without Catheter Ablation

**Figure 7:**
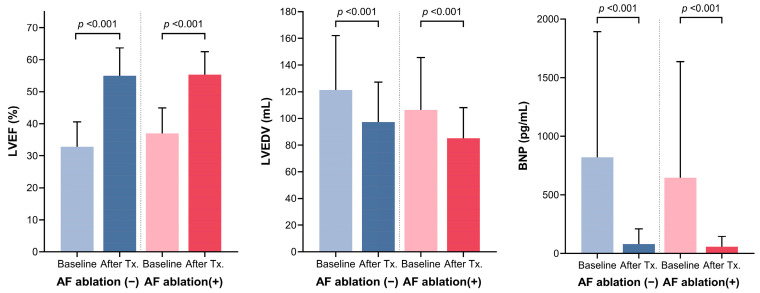
Change of echocardiographic parameter and brain natriuretic peptide (BNP) according to patients with or without catheter ablation. LVEF = left ventricular ejection fraction, LVEDV = left ventricular end-diastolic volume, Tx = therapy.

Table 2 shows the baseline characteristics of patients divided by whether they had catheter ablation or not. Patients who underwent catheter ablation had significantly lower BNP levels and higher LVEF compared to those who did not have the procedure. Regardless of catheter ablation, there was a significant improvement in LVEF, left ventricular end-diastolic volume, and BNP levels after rhythm control treatment (Figure 7). During the follow-up period, patients who had catheter ablation were much less likely to continue taking AADs compared to those who did not have catheter ablation (22 out of 30 [73.3%] vs. 48 out of 50 [96.0%], *p* value = 0.005).

## 4. Discussion

This study provides insight into the real-world outcomes of rhythm control therapy in patients with both AF and reduced LVEF. The main findings are as follows: (1) Sinus rhythm was restored in patients either through the use of AADs (38, 47.5%) or electrical cardioversion (42, 52.5%). (2) During the follow-up period of 53.0 ± 25.5 months, AF recurred in 52 patients (65.0%), with 30 patients (37.5%) undergoing catheter ablation. (3) There was a significant improvement of LVEF (from 34.3 ± 8.1% at baseline to 55.1 ± 8.1% at the last follow-up; *p* < 0.001) and a considerable reduction in BNP levels (from 752 ± 1039 pg/mL at baseline to 72 ± 114 pg/mL at the last follow-up; *p* < 0.001). (4) Nearly all patients (*n* = 78, 97.5%) continued with the rhythm control strategy, with only two patients (2.5%) switching to a rate control strategy during the follow-up period.

Several studies have compared changes in LVEF after rhythm control therapy versus rate control therapy in patients with AF and HF. In a randomized trial involving 61 patients with AF and LV systolic dysfunction, Shelton R.J. et al. observed a more significant improvement in LVEF at one year in patients assigned to the rhythm control with amiodarone compared with those in rate control group [18]. Further randomized trials have highlighted the efficacy of catheter ablation over pharmacological rhythm control in improving LVEF. Hunter R.J. et al. reported in a small randomized trial that LVEF was significantly higher in the catheter ablation group (40 ± 12%) than in the medical therapy group (31 ± 13%) after six months (*p* = 0.015) [19]. The CAMERA-MRI study, which focused exclusively on patients with potential AF-related cardiomyopathy evaluated by MRI (idiopathic cardiomyopathy with LVEF ≤ 45%), found that rhythm control therapy via catheter ablation significantly improved LVEF compared to rate control (mean difference 7.5%, *p* = 0.014) [20]. In the CASTLE-AF trial, which included patients with AF and LVEF ≤ 35%, the median absolute improvement in LVEF was 8.0% in the ablation group compared to 0.2% in the medical therapy group at the 60 months follow-up (*p* = 0.005) [15].

Though these randomized trials demonstrated the superiority of catheter ablation over drug therapy in rhythm control strategy for AF patients with reduced LVEF, they do not reflect real-world clinical practice. Catheter ablation might not be performed as the initial rhythm therapy for various reasons. Conversely, our study elucidates the effects of AADs or electrical cardioversion for sinus conversion in patients with AF and reduced LVEF, showcasing the feasibility and effectiveness of a long-term rhythm control strategy, including AADs and/or catheter ablation, depending on the recurrence of AF. Moreover, in response to these findings, recent guidelines for AF management have been updated to recommend catheter ablation for rhythm control as a class I indication in AF patients with HF with reduced LVEF [21]. However, at the time our study patients were treated for AF and HF, catheter ablation was not the recommended first choice for rhythm control. Additionally, while catheter ablation is recommended with class I evidence for rhythm control, it is not mandated as the first choice or a compulsory treatment for AF patients with reduced LVEF. Our results, primarily focusing on the recovery of LV function and changes in BNP, also support the superiority of a rhythm control strategy in AF patients with reduced LVEF. Moreover, patients with AF and severely reduced LVEF (<35%) may be perceived as a fragile patient group and could be considered at higher risk for catheter ablation. For these patients, a first attempt with AADs therapy and/or electrical cardioversion may appear safer as a first-line treatment. Additionally, AF is associated with an increased risk of HF. Recent findings suggest that fibrosis and structural atrial remodeling are pivotal in the development of AF in patients with HF [22]. Clinically, patients with AF and concurrent LVEF reduction exhibit worse outcomes, necessitating the prompt termination of AF to prevent irreversible electrical and mechanical remodeling of the LA.

Although improvement in LVEF is a positive outcome, it does not directly correlate with better clinical outcomes. Previous randomized trials assessing pharmacologic rhythm therapy in patients with AF and HF did not demonstrate a reduction in mortality compared to rate control therapy [11,12,13,23]. However, the CHF-STAT trial highlighted the significance of maintaining sinus rhythm, showing that it could significantly reduce mortality, despite the limitations of AADs in maintaining sinus rhythm for an extended period [23]. In four randomized trials, maintenance of sinus rhythm with AADs during the study’s follow-up period (ranging from 12 to 42 months) was achieved in only 30 to 40% of patients [11,12,13,23]. Consistent with prior research, our study found that sinus rhythm was maintained with AADs in only 35% of patients, and catheter ablation was required in 37.5% of patients to maintain sinus rhythm. Recent randomized trials have shown the efficacy of catheter ablation and/or AADs in reducing mortality and hospitalization for HF compared to rate control or pharmacologic rhythm control strategies [14,15,19,24,25]. These findings indicate that stricter rhythm control strategies may lead to better clinical outcomes and improved LV systolic function. Although our study did not specifically evaluate clinical outcomes such as cardiovascular death and hospitalization for HF, two instances of death and one instance of hospitalization for HF were observed during the follow-up period. Furthermore, there was no need for implantable cardioverter–defibrillator placement or cardiac resynchronization therapy.

In addition to improving LVEF, our study’s rhythm control strategy also demonstrated a significant reduction in BNP levels. BNP, a cardioprotective hormone released by cardiomyocytes in response to pressure or volume overloading [26], is a well-known marker indicating the severity and prognosis of HF [27]. Therefore, the significant decrease in BNP levels observed through rhythm control in our study indirectly supports the hypothesis that more effective management of AF and HF can lead to improved patient outcomes.

Several mechanisms have been proposed to explain the association between AF and HF. The major mechanism is believed to be tachycardia-mediated cardiomyopathy, which results from a rapid ventricular response during AF, leading to HF [28]. Another possible mechanism involves the loss of atrial contraction and irregular ventricular activity, which contributes to reduced cardiac output, further complicating HF beyond the effects of tachycardia-mediated cardiomyopathy [29,30,31]. Additionally, cardiac remodeling, along with the activation of neurohormonal and pro-fibrotic pathways, is promoted by elevated LA and LV filling pressure [32]. In the subgroup analysis of our study, we observed that the LVEF sequentially increased following GDMT for HF with rate control and sinus rhythm restoration. These findings underscore that the restoration of sinus rhythm may play a more pivotal role than rate control alone in the treatment of patients with concomitant AF and reduced LVEF. This supports the notion that strategies aiming to restore and maintain sinus rhythm could be beneficial in mitigating the cardiac dysfunction associated with AF and HF. Based on these findings, it should be considered that AF is not merely a secondary condition caused by HF, but rather a causal factor in the development of HF.

## 5. Limitations

This single-center, retrospective study included a small number of patients and had a single-arm design. Consequently, differentiating the specific benefits of rhythm control therapy beyond those of GDMT for HF is challenging, mainly due to the absence of a control group. This limitation hinders a clear evaluation of rhythm control therapy’s independent effect on improving LVEF and BNP levels without the simultaneous influence of optimal HF medical therapy. Moreover, a recent study for patients with AF and reduced LVEF reported that the group receiving optimally targeted GDMT had better survival rates compared to the non-targeted GDMT group [33]. Despite these constraints, our subgroup analysis of patients undergoing serial echocardiography showed a progressive improvement in LVEF following GDMT for HF and the subsequent adoption of a rhythm control strategy. This suggests that rhythm control may play a crucial role in enhancing LVEF alongside GDMT for HF. Given the strong evidence supporting catheter ablation as a class I first-line indication in patients with AF and HF, our results might be influenced by our management protocol for these patients. However, this may reflect a specific real-world scenario. In practice, nearly all patients were managed with a rhythm control strategy, despite only about one-third receiving catheter ablation during the follow-up period. Potential selection bias and variability in the timing of follow-up echocardiography and BNP measurements during follow-up could further limit our findings. However, our results demonstrated serial improvements in LVEF and BNP levels following the rhythm control strategy. Additionally, while this study did not primarily aim to assess cardiovascular outcomes such as cardiovascular death and hospitalization for HF, it is noteworthy that very few adverse cardiac outcomes were reported during the follow-up period. These findings could be explained by significant improvement of LVEF and BNP levels after rhythm control treatment. Finally, determining the exact cause of HF at the onset is challenging. The study focused on patients presenting with both AF and reduced LVEF at the onset, considering AF as a major contributing factor to the reduced LVEF. The strategy was to initiate and sustain rhythm control to mitigate the impact of AF-related cardiomyopathy. In instances where patients did not experience recovery of LVEF despite maintaining sinus rhythm, we diagnosed idiopathic dilated cardiomyopathy, although such cases were infrequent. Therefore, the reduced LVEF observed in the study patients may primarily result from the onset of AF. Our findings may be challenging to apply to patients with LV dysfunction due to other causes. Despite several limitations, this study offers insights into the real-world clinical practice and outcomes of rhythm control strategy in AF patients with reduced LVEF, including improvements in LVEF and BNP levels. Thus, these findings may provide valuable encouragement for physicians to consider active rhythm control therapy in managing these challenging patients.

## 6. Conclusions

In real-world practice, the adoption of an initial rhythm control strategy, integrating AADs and/or electrical cardioversion with GDMT for HF management, has proven both feasible and effective for achieving sinus rhythm in patients with concurrent AF and reduced LVEF. Despite AF recurrence in about two-thirds of cases, invasive catheter ablation was required for one-third during the follow-up period. The continuation of rhythm control in nearly all patients led to significant improvements in LVEF and BNP levels. These outcomes provide additional support for the efficacy of active rhythm control strategies in patients experiencing clinical deterioration.

## Figures and Tables

**Table 1:** Baseline characteristics of study patients.

	N = 80
Age, years	63.6 ± 10.9
Male sex, *n* (%)	65 (81.3)
Body mass index, kg/m^2^	25.4 ± 3.4
Atrial fibrillation type	
Paroxysmal	7 (8.8)
Persistent	58 (72.5)
Longstanding persistent	15 (18.8)
AF duration, days	273 ± 606
Hemoglobin, g/dL	14.6 ± 1.7
Creatinine, mg/dL	1.04 ± 0.58
BNP, pg/mL	789 ± 1133
Hypertension, *n* (%)	42 (52.5)
Diabetes, *n* (%)	25 (31.3)
Coronary artery disease, *n* (%)	5 (6.3)
Myocardial infarction, *n* (%)	1 (1.3)
PCI, *n* (%)	4 (5.0)
Stroke/TIA, *n* (%)	6 (7.5)
CHA_2_DS_2_VASc score, mean	2.4 ± 1.8
LVEF, %	34.3 ± 8.1
LVEF < 40%, *n* (%)	55 (68.8)
LVEF 40–50%, *n* (%)	25 (31.3)
LA diameter, cm	4.3 ± 0.6
LA volume index, mL/m^2^	41.8 ± 14.7
LVEDV, mL	115.6 ± 40.7
LVEDVI, mL/m^2^	62.4 ± 23.3
LVESV, mL	77.9 ± 34.0
LVESVI, mL/m^2^	41.9 ± 18.9
Mitral regurgitation grade	
None, *n* (%)	13 (16.3)
Minimal, *n* (%)	28 (35.0)
Mild, *n* (%)	25 (31.3)
Moderate, *n* (%)	14 (17.5)
Severe, *n* (%)	0
Tricuspid regurgitation grade	
None, *n* (%)	11 (13.8)
Minimal, *n* (%)	34 (42.5)
Mild, *n* (%)	25 (31.3)
Moderate, *n* (%)	5 (6.3)
Severe, *n* (%)	5 (6.3)
Beta-blocker, *n* (%)	74 (92.5)
ACEi/ARB, *n* (%)	64 (80.0)
MRA, *n* (%)	57 (71.3)
Digoxin, *n* (%)	24 (30.0)
Antiarrhythmic drugs, *n* (%)	80 (100.0)
Flecainide, *n* (%)	18 (22.5)
Propafenone, *n* (%)	3 (3.8)
Amiodarone, *n* (%)	53 (66.3)
Dronedarone, *n* (%)	1 (1.3)
Sotalol	5 (6.3)

ACEi = angiotensin-converting enzyme inhibitor; ARB = angiotensin receptor blocker; BNP = brain natriuretic peptide; CHA_2_DS_2_VASc score = congestive heart failure, hypertension, age ≥ 75 years, diabetes mellitus, prior stroke or transient ischemic attack or thromboembolism, vascular disease, age 65–74 years, sex category; LA = left atrium; LVEF = left ventricular ejection fraction; LVEDV = left ventricular end-diastolic volume; LVEDVI = left ventricular end-diastolic volume index; LVESV = left ventricular end-systolic volume; LVESVI = left ventricular end-systolic volume index; MRA = mineralocorticoid receptor antagonist; NDH-CCB = non-dihydropyridine calcium channel blocker; PCI = percutaneous coronary intervention; TIA = transient ischemic attack.

**Table 2:** Comparison of baseline characteristics between patients with or without catheter ablation.

	AF Ablation (−), N = 50	AF Ablation (+), N = 30	*p*-Value
Age, years	64.6 ± 11.6	62.0 ± 9.4	0.292
Male sex, *n* (%)	39 (78.0)	26 (86.7)	0.336
Body mass index, kg/m^2^	25.0 ± 3.7	26.1 ± 2.8	0.167
Atrial fibrillation type			0.356
Paroxysmal	5 (10.0)	2 (6.7)	
Persistent	38 (76.0)	20 (66.7)	
Longstanding persistent	7 (14.0)	8 (26.7)	
AF duration, days	212 ± 556	374 ± 679	0.250
Hemoglobin, g/dL	14.3 ± 1.8	15.1 ± 1.6	0.056
Creatinine, mg/dL	1.1 ± 0.7	1.0 ± 0.2	0.510
BNP, pg/mL	464 ± 426	238 ± 237	0.003
Hypertension, *n* (%)	27 (54.0)	15 (50.0)	0.729
Diabetes, *n* (%)	17 (34.0)	8 (26.7)	0.493
Coronary artery disease, *n* (%)	2	3	
Myocardial infarction, *n* (%)	0	1 (3.3)	0.375
PCI, *n* (%)	2 (4.0)	2 (6.7)	0.628
Stroke/TIA, *n* (%)	6 (12.0)	0	0.079
CHA_2_DS_2_VASc score, mean	2.6 ± 2.0	2.1 ± 1.3	0.169
LVEF, %	32.8 ± 7.8	36.9 ± 8.0	0.025
LVEF < 40%, *n* (%)	40 (80.0)	15 (50.0)	0.005
LVEF 40–50%, *n* (%)	10 (20.0)	15 (50.0)	
LA diameter, cm	4.6 ± 0.7	4.6 ± 0.5	0.812
LA volume index, mL/m^2^	51.8 ± 15.4	49.0 ± 14.8	0.452
LVEDV, mL	121.3 ± 40.8	106.4 ± 39.4	0.115
LVEDVI, mL/m^2^	65.2 ± 25.1	57.6 ± 19.4	0.161
LVESV, mL	83.5 ± 34.0	68.9 ± 32.8	0.065
LVESVI, mL/m^2^	44.7 ± 19.8	37.3 ± 16.6	0.087
Mitral regurgitation grade			
None, *n* (%)	8 (16.0)	5 (16.7)	
Minimal, *n* (%)	17 (34.0)	11 (36.7)	
Mild, *n* (%)	14 (28.0)	11 (36.7)	
Moderate, *n* (%)	11 (22.0)	3 (10.0)	
Severe, *n* (%)	0	0	
Tricuspid regurgitation grade			
None, *n* (%)	8 (16.0)	3 (10.0)	
Minimal, *n* (%)	20 (40.0)	14 (46.7)	
Mild, *n* (%)	14 (28.0)	11 (36.7)	
Moderate, *n* (%)	5 (10.0)	0	
Severe, *n* (%)	3 (6.0)	2 (6.7)	
Beta-blocker, *n* (%)	47 (94.0)	27 (90.0)	0.667
ACEi/ARB, *n* (%)	40 (80.0)	24 (80.0)	1.000
MRA, *n* (%)	38 (76.0)	19 (63.3)	0.226
Digoxin, *n* (%)	13 (26.0)	11 (36.7)	0.313
Antiarrhythmic drugs, *n* (%)	50 (100)	30 (100)	
Flecainide, *n* (%)	9 (18.0)	9 (30.0)	
Propafenone, *n* (%)	1 (2.0)	2 (6.7)	
Amiodarone, *n* (%)	38 (76.0)	15 (50.0)	
Dronedarone	1 (2.0)	0	
Sotalol	1 (2.0)	4 (13.3)	

ACEi = angiotensin-converting enzyme inhibitor; ARB = angiotensin receptor blocker; BNP = brain natriuretic peptide; CHA_2_DS_2_VASc score = congestive heart failure, hypertension, age ≥ 75 years, diabetes mellitus, prior stroke or transient ischemic attack or thromboembolism, vascular disease, age 65–74 years, sex category; LA = left atrium; LVEF = left ventricular ejection fraction; LVEDV = left ventricular end-diastolic volume; LVEDVI = left ventricular end-diastolic volume index; LVESV = left ventricular end-systolic volume; LVESVI = left ventricular end-systolic volume index; MRA = mineralocorticoid receptor antagonist; PCI = percutaneous coronary intervention; TIA = transient ischemic attack.

## Data Availability

Data that support the findings of this study are available on request from the corresponding author. The data are not available publicly due to privacy or ethical restrictions.

## Abbreviations

AADs	anti-arrhythmic drugs
AF	atrial fibrillation
BNP	brain natriuretic peptide
GDMT	guideline-directed medical therapy
HF	heart failure
LA	left atrium
LVEF	left ventricular ejection fraction
